# Supplementary material for: In Dormant Red Rice Seeds, the Inhibition of Early Seedling Growth, but Not of Germination, Requires Extracellular ABA
Source: Plants (Basel). 2022 Apr 9;11(8):1023. doi: 10.3390/plants11081023 (PMC9025618; doi:10.3390/plants11081023)
Supplement: Supplementary file 1 [file plants-11-01023-s001.zip › Supplementary Figure S3.pdf]

Supplementary Figure S3

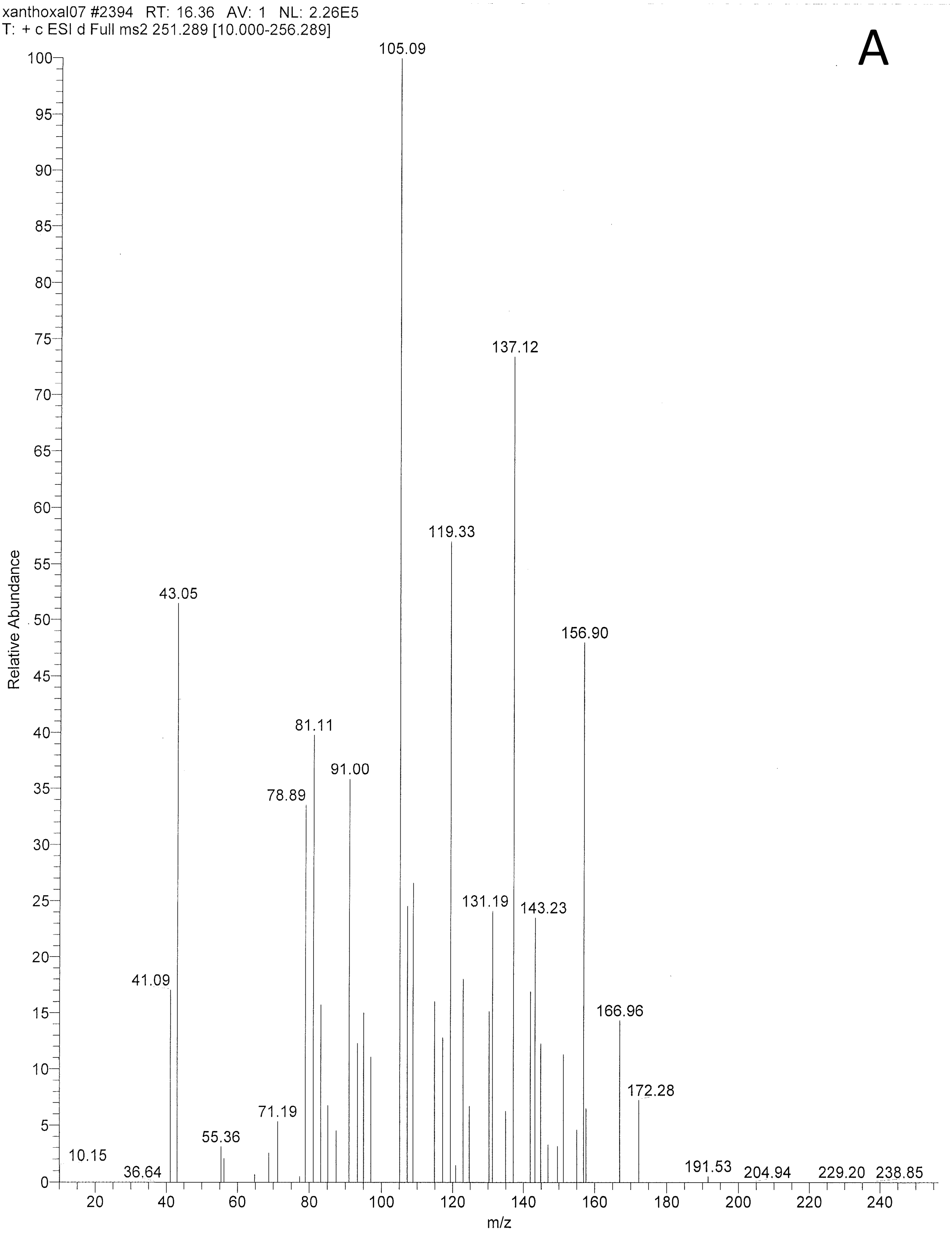

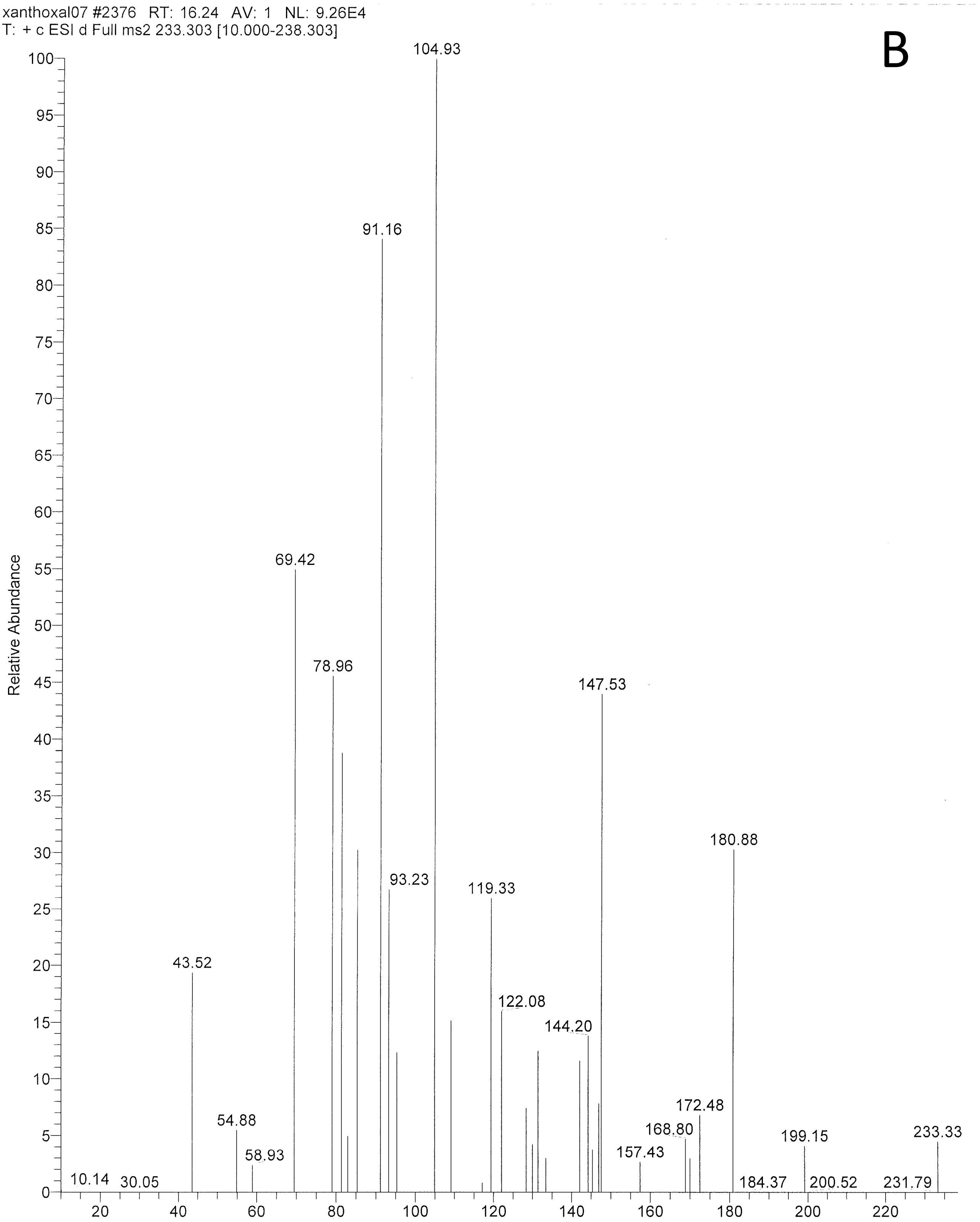

Supplementary Figure S3. LC-ESI-MS/MS fragmentation analysis (at 30 eV) of the molecules corresponding to the two main peaks of Figure 3: (**A**) fragmentation of the molecular peak with m/z 251, and (**B**) fragmentation of the dehydrated molecule with m/z 233. Although the fragmentation pattern is quite complex, the presence of peaks with m/z 105 and 91 is evident, particularly for the dehydrated molecule. They should correspond to the stable ions methyl tropylium (MW 105.16) and tropylium (MW 91.13), respectively, which are typically formed in ESI$^+$ by split and rearrangement of a molecule whose skeleton consists of a benzene (or a six-carbon ring that can rearrange to a benzene) with a side chain. This is, indeed, consistent with the xanthoxal structure.
