# Supplementary material for: In Dormant Red Rice Seeds, the Inhibition of Early Seedling Growth, but Not of Germination, Requires Extracellular ABA
Source: Plants (Basel). 2022 Apr 9;11(8):1023. doi: 10.3390/plants11081023 (PMC9025618; doi:10.3390/plants11081023)
Supplement: Supplementary file 1 [file plants-11-01023-s001.zip › Supplementary Figure S4.pdf]

Supplementary Figure S4

A

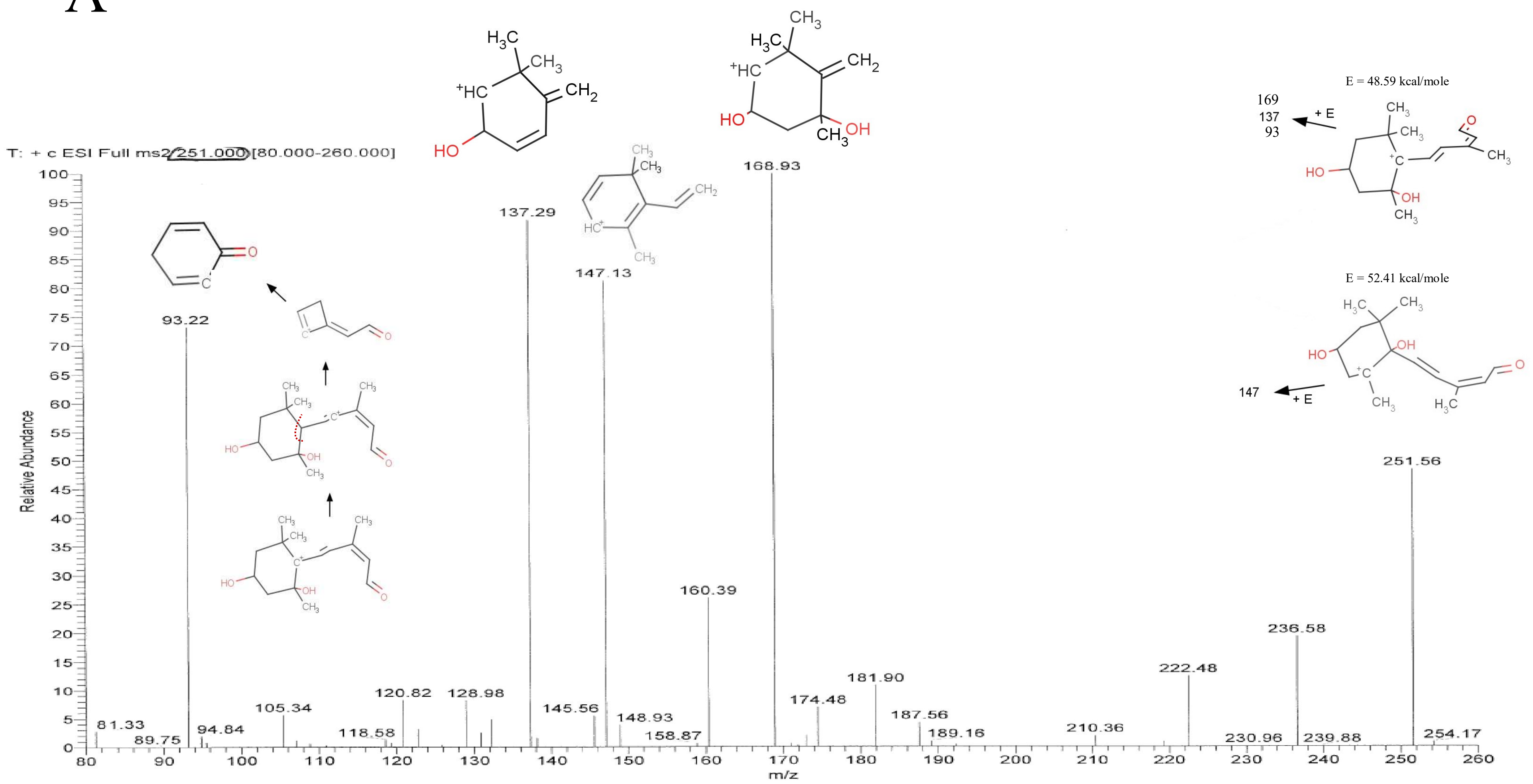

B

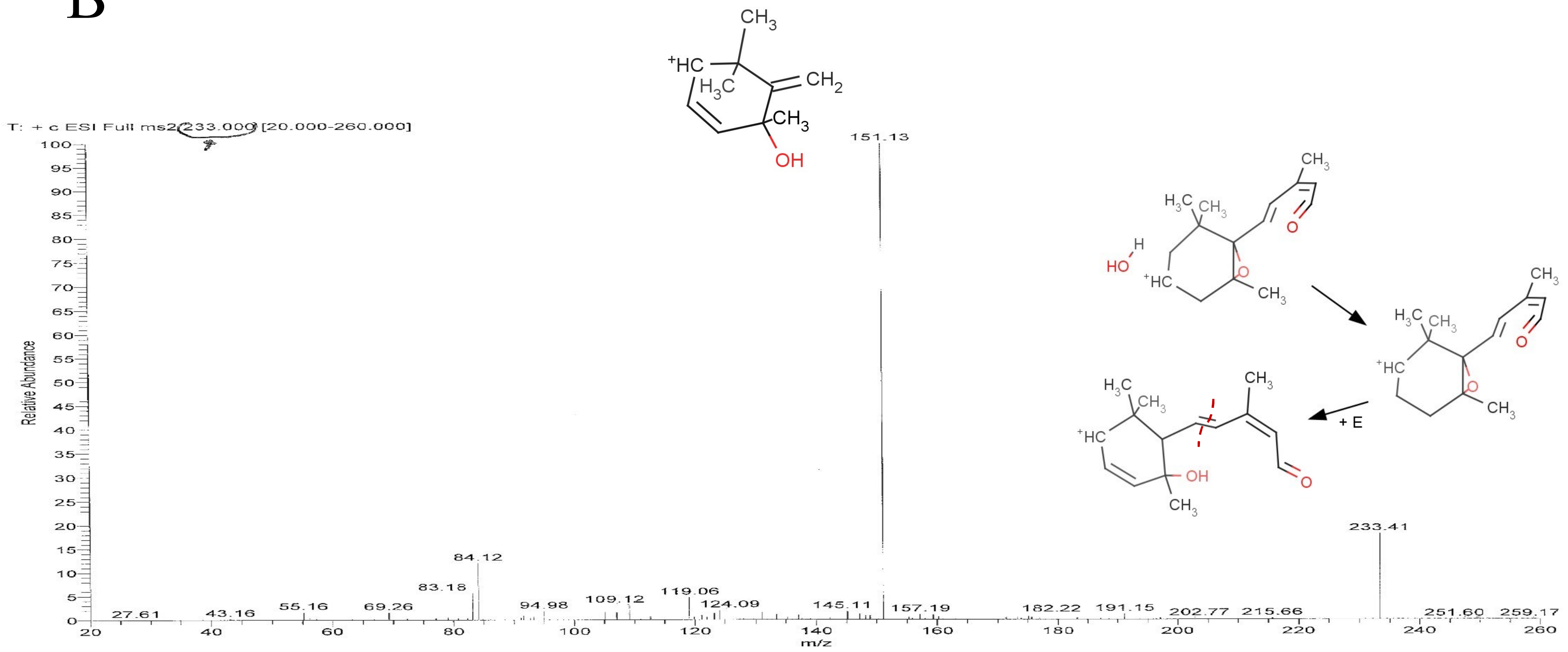

Supplementary Figure S4. Direct infusion ESI-MS/MS ("soft") fragmentation analysis of the protonated molecules of the two main peaks of Figure 3: (**A**) fragmentation of the molecular peak at m/z 251, and (**B**) fragmentation of the dehydrated molecular ion with m/z 233. The putative ion structure is shown for major peaks (referred to the *cis*,*trans*-xanthoxal isomer). The values of the Dreiding energy of the two tertiary carbocations of the xanthoxal molecular ion (as from Figure 3), and the possible genesis of the main fragments from them, are illustrated in (A). In general, when a molecule consists in a ring with a relatively large side chain, "soft" collision-induced dissociation usually splits the side chain from the ring, as the former levers on the latter. Commonly, the carbocation is retained within the ring because of greater stability, and, therefore, the side chain does not produce detectable fragments. Nonetheless, the peak at m/z 93 might derive from the side chain, starting from the more stable carbocation (at m/z 251), if the charge were transferred to the side chain (as illustrated in A), by resonance (albeit transitorily, since it would be a less stable secondary carbocation) or as a consequence of the collision that split the molecular ion, and this was followed by the cleavage of the single bond between the ring and the first carbon of the side chain next to the ring. The broken bond at the side-chain carbon previously linked to the ring would then re-form with the primary carbon on the branch of the chain, thereby promptly balancing the hydrogens. This would lead to

cyclization and formation of a tertiary carbocation. Further collisions could cause the four-carbon ring (which has non-negligible steric strain) to re-arrange to the oxocyclohexadienyl (or phenoxy) cation (as shown in A, but many resonance forms are possible). The structure assumed, in (A), for the fragment with m/z 147 was the same as that obtained by ESI+ retro-ene fragmentation of astaxanthin and prasinoxanthin (Neto et al. 2016, Rapid Commun. Mass Spectrom. 30:1540), carotenoids whose ring structure is similar to that of the carbocations of the xanthoxal molecular peak illustrated in (A). This fragment would be produced through elimination of part of the side chain and of two water molecules. It is supposed that this fragment could originate from the less stable of the two molecular carbocations (the one with the higher Dreiding energy), because this latter allows an easier shift of the charge to secondary carbons in the ring, from which hydrogens can be drawn to carry out the splitting of the molecular ion and the elimination of the hydroxyl group, derived from the epoxide, as water. The fragmentation pattern of the dehydrated molecular ion, shown in (B), was much simpler: low-energy collisions essentially produced a single fragment with m/z 151, owing to the loss of the side chain. Although the split might be supposed to occur at the single bond between the ring and the first carbon of the side chain next to the ring, it rather took place between the first and second carbon atoms of the side chain next to the ring, thereby breaking a (stronger) double bond (like observed for the molecular peak with m/z 251). In fact, breaking the single bond joining the side chain to the ring would require the formation of a new bond for the involved carbon of the ring, but the two carbon atoms adjacent to it in the ring are already fully substituted. Instead, the break of the double bond closer to the ring can be balanced by the transformation of the single bond between the ring and the first carbon of the side chain next to the ring into a double bond, with a simple transfer of one hydrogen atom from the carbon of the ring to what was the first carbon of the side chain. This would not be possible if the epoxide were intact. Thus, it can be inferred that the collision that cleaved the dehydrated molecular ion also provided the energy required to open the epoxide. The simultaneity of the cleavage of the side chain and the opening of the epoxide would therefore allow the hydroxy group to form only at the position where it has been drawn in (B), differently from the molecular ion, in which the hydroxyl group can end up on one or the other of the two carbon atoms previously involved in the epoxide group, as shown in (A). A fragment like that with m/z 147, obtained in (A) from the molecular ion, could not, therefore, form from the dehydrated molecular ion. As regards the structure of the fragments at m/z 169 and 137 observed in (A), it was inferred based on the simplest provision of the hydrogens required for a re-arrangement of the ring following a molecular split as described for the fragment with m/z 151 observed in (B). Indeed, in (A), the structure assumed for the peak at m/z 169 is just the hydrated form of the fragment with m/z 151 in (B), and the structure at m/z 137 (which was observed also in the fragmentation at high energy; Supplementary Figure 1) would be obtained by elimination of a methanol molecule from the fragment with m/z 169. A similar elimination is prevented in the fragment with m/z 151, in (B), because a double bond is already present in the ring and no other double bond can form. Besides, in (B), the peak at m/z 93 is absent because the described mechanism following the simultaneous splitting of the molecular ion and the opening of the epoxide would prevent its formation, since the charge could not be transferred to the side chain. For illustrative purposes, the arrangements to the ring that presumably lead to the fragment with m/z 151, in (B), are represented for the whole dehydrate molecular ion, neglecting the concomitant cleavage of the side chain. Ultimately, the fragmentation patterns of the molecular peak and its dehydrated form appear entirely consistent with the assumption that the analyzed molecule is xanthoxal.
