# Supplementary material for: In Dormant Red Rice Seeds, the Inhibition of Early Seedling Growth, but Not of Germination, Requires Extracellular ABA
Source: Plants (Basel). 2022 Apr 9;11(8):1023. doi: 10.3390/plants11081023 (PMC9025618; doi:10.3390/plants11081023)
Supplement: Supplementary file 1 [file plants-11-01023-s001.zip › Supplementary Figure S5.pdf]

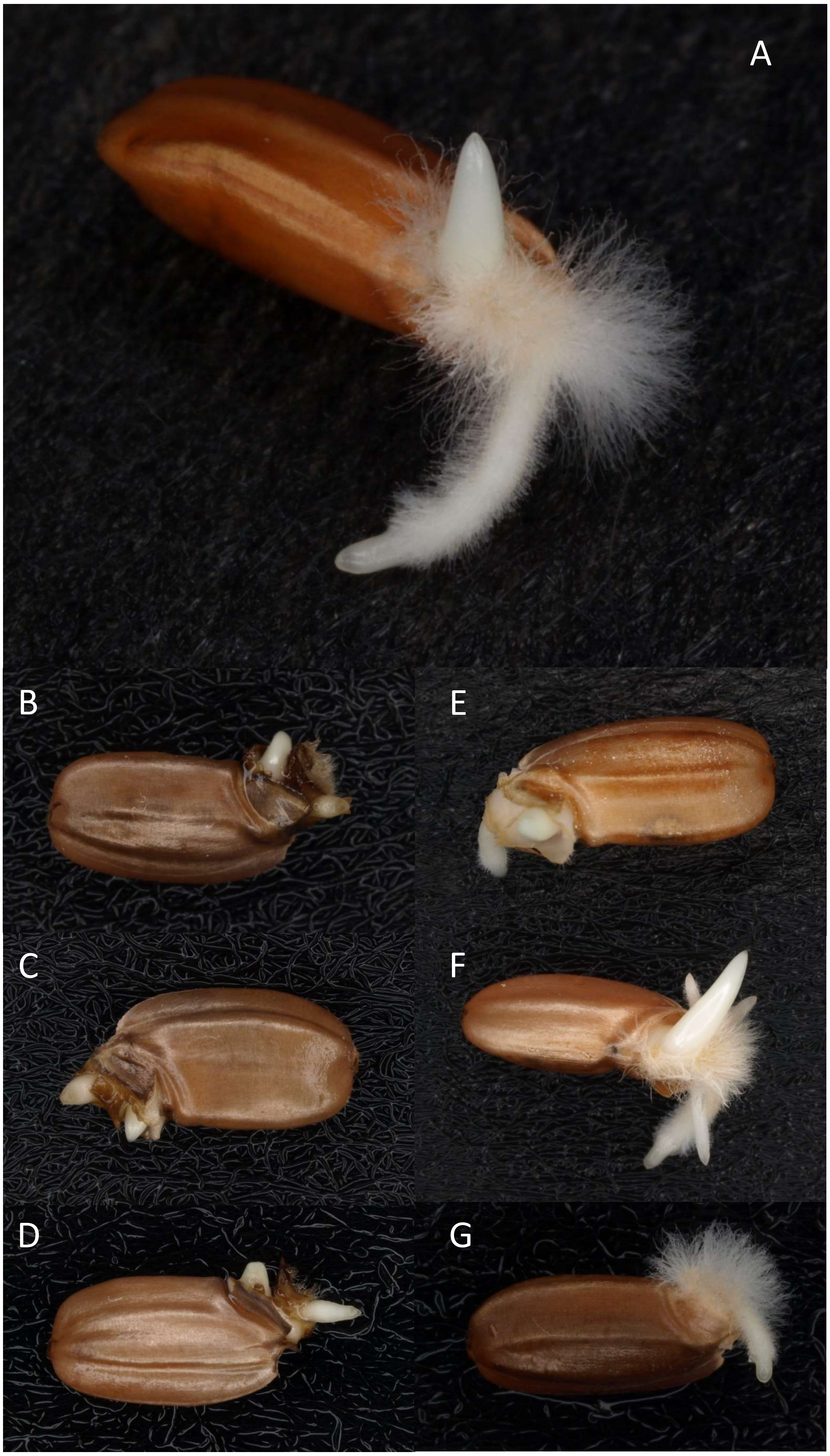

Supplementary Figure S5. Aspect of the embryo collar and development of rhizoids during early seedling growth in dormant red rice seeds treated with: (**A**) fluridone, (**B-D**) fluridone plus ABA, (**E-G**) fluridone plus xanthoxal. Seeds were assessed for germination (pericarp splitting) and early seedling growth (either radicle or coleoptile ≥ 1mm) thrice a week, photographs were taken when the latter stage was observed. Three seedlings are shown for both fluridone plus ABA and fluridone plus xanthoxal to show that, though in the former condition the seedlings had a quite uniform aspect, under the latter condition there was greater variability, but the embryo collar typically did not display a necrotic aspect.
