# Supplementary material for: In Dormant Red Rice Seeds, the Inhibition of Early Seedling Growth, but Not of Germination, Requires Extracellular ABA
Source: Plants (Basel). 2022 Apr 9;11(8):1023. doi: 10.3390/plants11081023 (PMC9025618; doi:10.3390/plants11081023)
Supplement: Supplementary file 1 [file plants-11-01023-s001.zip › Supplementary Figure S1.pdf]

Supplementary Figure S1

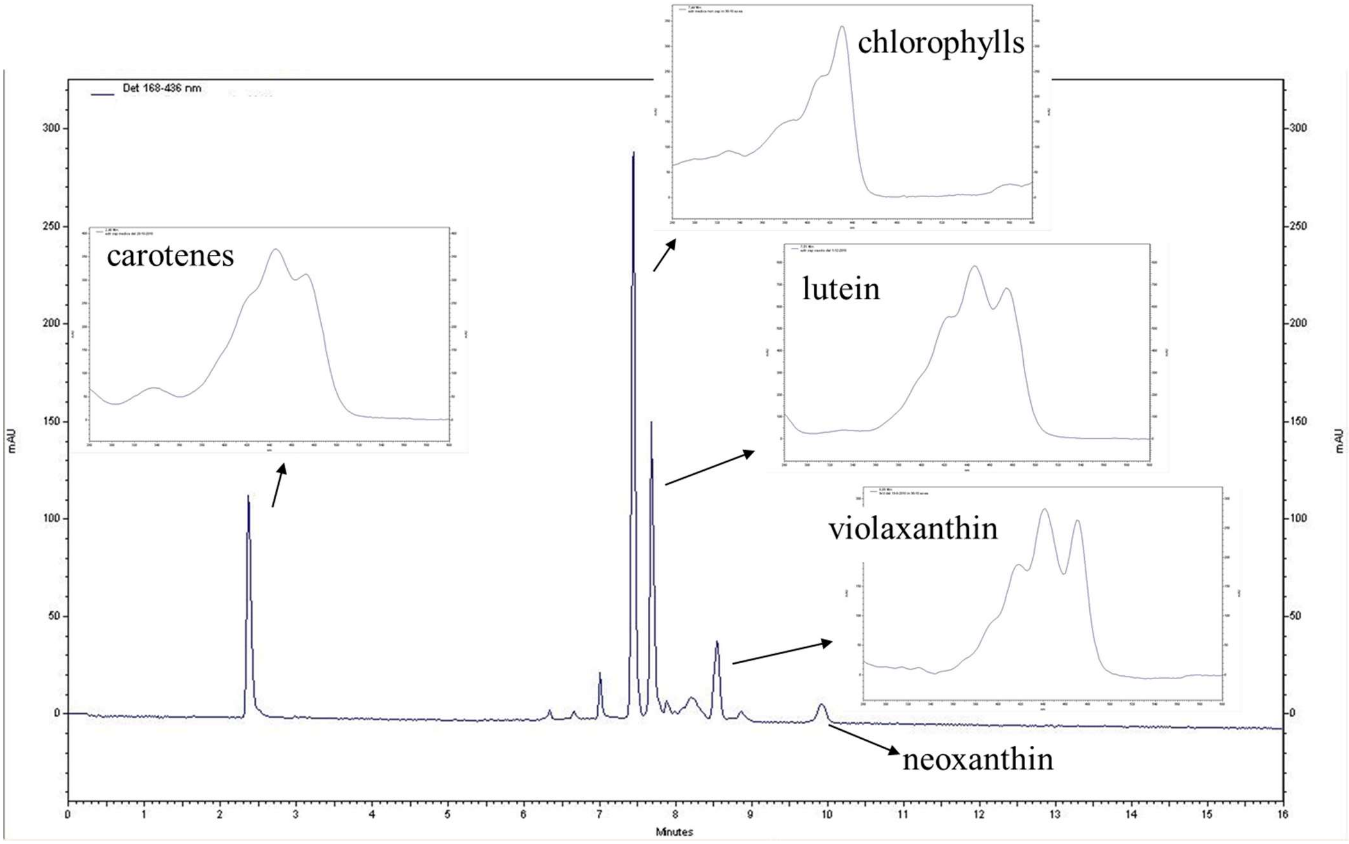

Supplementary Figure S1. HPLC analysis of the raw extract in 90:10 hexane/ethyl acetate.
